# Malaria in South America: a drug discovery perspective

**DOI:** 10.1186/1475-2875-12-168

**Published:** 2013-05-24

**Authors:** Luiza R Cruz, Thomas Spangenberg, Marcus VG Lacerda, Timothy NC Wells

**Affiliations:** 1 Medicines for Malaria Venture, 20 route de Pré-Bois, Geneva, CH 1215, Switzerland; 2 Fundação de Medicina Tropical Dr Heitor Vieira Dourado, Av Pedro Teixeira, 25, Manaus, Amazonas, 69040-000, Brazil

**Keywords:** South America, Malaria, *Plasmodium*, *Plasmodium vivax*, Treatment, Resistance, Drug discovery, Review

## Abstract

The challenge of controlling and eventually eradicating malaria means that new tools are urgently needed. South America’s role in this fight spans both ends of the research and development spectrum: both as a continent capable of discovering and developing new medicines, and also as a continent with significant numbers of malaria patients. This article reviews the contribution of groups in the South American continent to the research and development of new medicines over the last decade. Therefore, the current situation of research targeting malaria control and eradication is discussed, including endemicity, geographical distribution, treatment, drug-resistance and diagnosis. This sets the scene for a review of efforts within South America to discover and optimize compounds with anti-malarial activity.

## Background

Malaria is the tropical disease with the highest global mortality. In 2010, there were an estimated 216 million cases of malaria and 655,000 deaths worldwide, with children under five years and pregnant women the most vulnerable [1]. Over 81% of cases and 91% of deaths were in Africa, with the majority of the remaining being in India, Southeast Asia and South America.

South America includes 13 countries: Argentina, Bolivia, Brazil, Chile, Colombia, Ecuador, Guyana, Paraguay, Peru, Suriname, Uruguay, Venezuela and French Guiana. Most malaria cases are concentrated in the Amazon basin, with 580,000 reported in 2010, mainly in Brazil (281,586) [2] and Colombia (115,000) [3]. In 2010 only 240 deaths were registered, 0.085% of the global total. This low number reflected a combination of factors: the higher quality of health care, and the fact that the majority of cases are *Plasmodium vivax* rather than *Plasmodium falciparum* (estimated in 70%). *Plasmodium vivax* mortality is often assigned to sequelae, such as haemolysis or lung inflammation, rather than the parasite itself [4,5]. Other species of malaria have been reported. Suriname [6] and French Guiana [7] report 12% and 6% *Plasmodium malariae* infections respectively, although this may be an underestimate resulting from difficult diagnosis in thick-smear blood or rapid tests.

Malaria has been a long-term health issue in South America. Throughout the 20th Century, the continent underwent a rapid and disorganized development and settlement process, leading to a population migration. In the Amazon basin, with increased prospecting for minerals and agricultural projects [8,9], work opportunities surged. This led to an increase in malaria prevalence and incidence in the 1970s and 1980s [10], a trend that is only now starting to be reversed [11].

South America, with its large biodiversity, has also played a key role in the identification of new medicines to combat malaria. The active cinchona bark, which led to the purification of quinine was first identified in Peru [12], and lapachol, the forerunner of atovaquone, also came from the Amazon basin [13]. This raises the question as to whether there are other natural products that could be useful in malaria. In addition, South America has an excellent scientific and clinical base, which can continue to support the discovery and development of new therapeutics. This review provides an overview of malaria in South America, focusing on progress in drug discovery, and highlighting critical future areas where the continent can support the malaria eradication agenda.

## Malaria in South America

**Figure 1:**
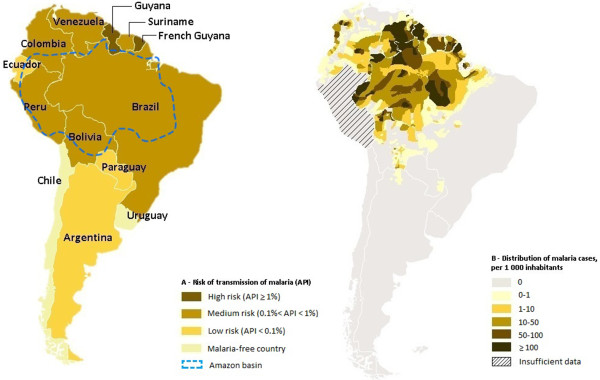
**Incidence and risk of transmission of malaria. A**- Risk of transmission of malaria, classified by country, in 2010. The dashed blue lines delimit the Amazon basin. **B**- Distribution of malaria cases in the Amazon basin, in 2010 (based on the WHO World Malaria Report 2011).

The endemicity of malaria can be divided into three levels: high risk, if the annual parasite incidence (API) is higher than 1% of the inhabitants; medium risk, when it is 0.1 to 1% and low risk where it is less than 0.1% [11], (see Figure 1).

Of all the South American countries, Uruguay and Chile are malaria free, with no mosquito-transmitted infections. Argentina and Paraguay are progressing towards elimination [1]. The remainder of the continent shows a broad distribution of cases, with increasing frequency towards the tropics. Brazil, has an overall API of 0.16%, reaching 0.6 to 0.7% in Amazonas and Acre [2]. On the other hand, in Colombia and Suriname 15% of the population live in areas with high transmission, and this number reaches 85% in French Guiana and Guyana where APIs of 35% have been reported locally [7].

In the rainforest region, the primary vector species that transmits *Plasmodium* parasites is *Anopheles darlingi*[14-16], with other species such as *Anopheles albitarsis*, *Anopheles albimanus*, *Anopheles aquasalis* and *Anopheles marajoara* playing roles in transmission [17-19]. *Anopheles gambiae* was imported into South America from Africa in the transatlantic slave trade but was eliminated from the continent in the first half of the 20th Century [20,21]. *Anopheles darlingi* is an efficient vector, preferring humans over animals, and with a high susceptibility to *Plasmodium* infection [16]. Although nets are important, they are not sufficient, since many vectors have peak-biting hours before bedtime [22,23] and in addition not all families have appropriate numbers of bed nets.

**Figure 2:**
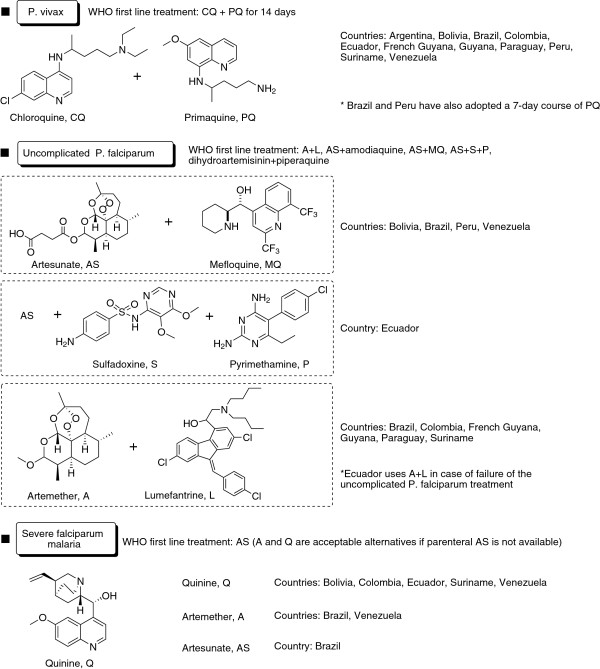
**Malaria treatment.** Standard treatment of malaria in South America according to the Ministries of Health from each country and to WHO guidelines for the treatment of various forms of malaria. There is also severe *vivax* malaria, for which the treatment should be similar to that used in the treatment of severe malaria caused by *Plasmodium falciparum*.

The standard treatment for uncomplicated *P. falciparum* malaria is artemisinin-based combination therapy (ACT), as recommended by the World Health Organization (WHO) [24], outlined in Figure 2. Chloroquine (CQ) is still effective for *P. vivax* in many countries. However, the Amazon Network for the Surveillance of Antimalarial Drugs Resistance (RAVREDA, Red Amazónica de Vigilancia de la Resistencia a los Antimaláricos) reported 10% resistance to chloroquine in Amazonas, Brazil [25]. Primaquine is the standard therapy for preventing relapses of *P. vivax*, although there are issues with compliance to the 14-day regimen and a risk of haemolysis in G6PD-deficient subjects. Studies in Brazil showed that primaquine failed to prevent relapses in 24.5% of cases [26]. Whether this is true resistance to the drug or lack of compliance is not clear. It has been suggested that an increased dosage of primaquine is required for South America [27], and Brazil and Peru have recently shifted from 14 days of 0.25 mg/kg/day to seven days of 0.5 mg/kg/day. G6PD deficiency was detected at 3% prevalence in Manaus, Brazil [28] and was predominantly the mild A^-^ form. In Buenaventura, Colombia, where a higher proportion of the population has African origins, prevalence of the mild A^-^ form is 12% [29]. An improved version of primaquine, developed originally by the Walter Reed Army Institute of Research (WRAIR), called tafenoquine, is under clinical development as a single-dose anti-relapse agent, but this is not expected to be launched before 2017. For severe falciparum malaria, most countries use parenteral quinine, although data from Africa and Asia support a shift to artesunate for injection [30], which has already been pioneered by Brazil.

### Search methodology

A literature search was conducted in February and March 2012 to identify studies regarding malaria research activities in South America. The sources for published data were SciFinder Scholar®, PubMed® and LILACS®. The date of publication considered spanned from January 2000 to February 2012. The following key words were used for the database search: malaria or anti-malarial. The search list was refined by country by means of the affiliation field. All papers describing any type of drug (based on medicinal chemistry, natural products or other approaches) were selected. Only research showing either *in vitro* or *in vivo* activities of molecules was considered. In addition, the database Thomson Pharma® was screened for clinical trials’ protocols conducted within the continent. Papers regarding drug discovery research were divided into two groups: those covering natural products (divided into plant extracts and isolated substances) and those covering studies of new synthetic drug compounds.

## Results

### Natural products

**Figure 3:**
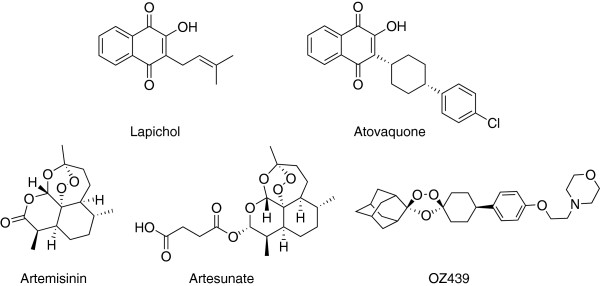
**Structures of lapachol, atovaquone, artemisinin, artesunate and OZ439.** Lapachol and artemisinin are examples of plant-derived compounds that originated anti-malarial compounds. Changes on these compounds gave atovaquone and artesunate, respectively. Additionally, OZ439 was inspired by artemisinin.

Pharmacognosy is the study of naturally occurring molecules with medicinal properties. Plant-derived compounds have been the backbone of the anti-malarial class of drugs over the last centuries, and two emerged from South America. Quinine is the active ingredient in cinchona tree bark in Peru and was purified in 1820, becoming the first disclosed compound with known anti-malarial activity. Lapachol, belonging to the chemical class of naphthoquinones, was first isolated from *Tabebuia avellanedae* in 1882 and used to treat fever and malaria in the 19th Century in South America. A third natural product, artemisinin, was isolated by Chinese scientists from *Artemisia annua.* These natural products have served as starting points for medicinal chemistry optimization. Chloroquine was designed based on quinine, massively reducing the frequency of administration, and paving the way for a whole new generation of aminoquinolines and amino-alcohols. The chemical optimization from lapachol to atovaquone gave new molecules with more reliable oral bio-availability, allowing them to be used in prophylaxis. Modifying artemisinin to artesunate massively improved solubility (Figure 3), but has also led to the design of new improved endoperoxides such as OZ439 which is currently in Phase II trial to evaluate its efficacy and stability in malaria patients [31]. These improved molecules have been used to treat hundreds of millions of patients over the last century.

**Figure 4:**
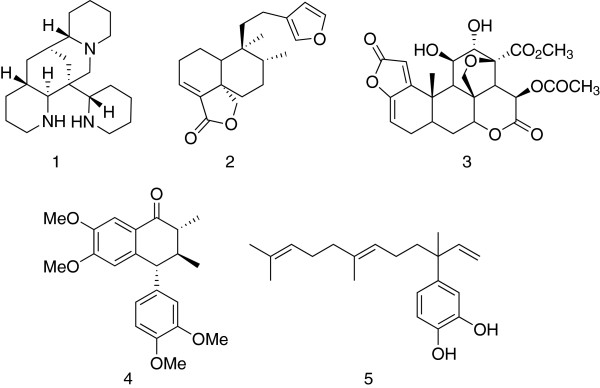
**Structures of plant-derived compounds.** These compounds show activities (EC_50_) below 1 μM.

South America has a long tradition of studies of natural products based on two approaches: the biological evaluation of traditional medicines and the identification of plants (or organisms) with differences in secondary metabolism [32]. The natural products identified (pharmacognosy) are shown on Table 1. A cut-off of approximately EC_50_ of 1 μg/mL (1 μM where the active ingredient is well characterized) was used based on the experience that almost 0.5% of chemical diversity is active at this level [33]. The structures of molecules are shown in Figure 4.

**Table 1:** Summary of pharmacognosy studies

**Compound**	**Authors**	**Plant with the lowest IC**_**50**_	**Type**	**IC**_**50 **_**(*P. falciparum *strain)**
**1**	**Bravo *et al.***[34]	***Bowdichia virgiloides***	**Extracts**	**1.0 μg/mL**
			**Isolated substances**	**5 μg/mL (F32 and Indo)**
**-**	**Deharo *et al.***[35]	***Bowdichia virgiloides***	**Extracts**	**1 μg/mL (F32)**
**2**	**da Silva Filho *et al.***[36]	***Baccharis dracunculifolia***	**Extracts**	**13 μg/mL**
			**Isolated substances**	**0.8 μg/mL (W2 e D6)**
**3**	**de Andrade-Neto *et al.***[37]	***Picrolemma spruce***	**Isolated substances**	**0.002 μM (K1)**
**4**	**de Andrade-Neto *et al.***[38]	***Holostylis reniformis***	**Isolated substances**	**0.20 μM (field isolate)**
**-**	**Kayano *et al.***[39]	***Caesalpina pluviosa***	**Extracts**	**0.59 μg/mL (3D7)**
**5**	**Rocha e Silva *et al.***[40]	***Piper peltatum***	**Isolated substances**	**0.05–2.11 μg/mL (M1)**
**5**	**Pinto *et al.***[41]	***Pothomorphe peltata***	**Isolated substances**	**0.67 μM (K1)**
**-**	**Garavito *et al.***[42]	***Remijia peruviana***	**Extracts**	**0.85 μg/mL (FcB2)**
**-**	**Debenedetti *et al.***[43]	***Buddleja globosa***	**Extracts**	**8.9 μg/mL (K1)**
**-**	**Baelmans *et al.***[44]	***Caesalpina pluviosa***	**Extracts**	**8 μg/mL (D2)**
**-**	**Flores *et al.***[45]	***Caesalpina pluviosa***	**Extracts**	**3.4 μg/mL**
			**Isolated substances**	**Inactive (F32)**
**-**	**Ibáñez-Calero *et al.***[46]	***Rumex obtusifolius***	**Isolated substances**	**71 μg/mL**^**i**^
**-**	**Muñoz *et al.***[47]	***Sparanttanthelium amazonum***	**Extracts**	**2 μg/mL (F32)**
**-**	**Muñoz *et al.***[48]	***Swietenia macrophylla***	**Extracts**	**73%**^**ii**^
**-**	**Muñoz *et al.***[49]	***Tripodanthus acutifolis***	**Extracts**	**98%**^**iii**^
**-**	**Costa *et al.***[50]	***Montrichardia linifera***	**Extracts**	**11.7 μg/mL (W2)**
**-**	**da Silva Filho *et al.***[51]	***Nectandra megapotamica***	**Extracts**	**28 μg/mL**
			**Isolated substances**	**3.8 μg/mL (D6)**
**-**	**de Andrade-Neto *et al.***[52]	***Bidens pilosa***	**Extracts**	**3.1 μg/mL (D6)**
**-**	**de Andrade-Neto *et al.***[53]	***Remijia ferruginea***	**Extracts**	**48%**^**iv**^
**-**	**de Mesquita *et al.***[54]	***Matayba guianensis***	**Isolated substances**	**2.5 μg/mL (FcB1)**
**-**	**Dolabela *et al.***[55]	***Esenbeckia febrifuga***	**Extracts**	**15.5 μg/mL**
			**Isolated substances**	**75.3 μg/mL (W2)**
**-**	**Estevam *et al.***[56]	***Ouratea nitida***	**Extracts**	**51.04%**^**v**^
**-**	**Fischer *et al.***[57]	***Xylopia emarginata***	**Extracts**	**3.3 μg/mL (PA)**
**-**	**Morais *et al.***[58]	***Pentacalia desiderabilis***	**Isolated substances**	**7.82 μg/mL (K1)**
**-**	**Oliveira *et al.***[59]	***Bidens pilosa***	**Extracts**	**38%**^**vi**^
**-**	**Sá *et al.***[60]	***Physalis angulata***	**Isolated substances**	**2.2 μM (W2)**
**-**	**Uchôa *et al.***[61]	***Cecropia pachystachya***	**Extracts**	**66%**^**4**^
			**Isolated substances**	**58%**^**vii**^
**-**	**Loyola *et al.***[62]	***Azorella compacta***	**Isolated substances**	**60%**^**viii**^
**-**	**Pabón *et al.***[63]	***Solanum nudum***	**Isolated substances**	**21 μM (FcB2)**
**-**	**Céline *et al.***[64]	***Siparuna aspera***	**Extracts**	**6.4 μg/mL (FCR-3)**
**-**	**Ruiz *et al.***[65]	***Minquartia guianensis***	**Extracts**	**4.2 μg/mL (FCR-3)**

^i^ Inhibition of biocrystallization of ferriprotoporphyrin IX.

^ii^ Percentage of inhibition of parasite growing (dose: 250 mg/kg).

^iii^ Percentage of inhibition of parasite growing (at 10 μg/mL).

^iv^ Reduction of parasitaemia (dose: 1000 mg/kg).

^v^ Activity tested against *Plasmodium berghei* in mice (dose: 1000 mg/kg).

^vi^ Reduction of parasitaemia at day 5 (dose: 250 mg/kg).

^vii^ Percentage inhibition of parasitaemia in relation to untreated infected mice on day 8 after malaria infection (doses: 250 and 15 mg/kg respectively).

^viii^ Activity in mice measured by the growth of inhibition (dose: 10 mg/kg/day).

Thus, only five purified compounds (Figure 4) have been identified from these efforts. Studies of *Bowdichia virgiloides,* a plant used by the Tacana indigenous group as a traditional medicine for the relief of high fever, produced alkaloid 1 (ormosanine), having an EC_50_ = 5 μg/mL against F32*. In vivo,* the extract showed 51% suppression of parasites in mice at 100 mg/kg, but was toxic at 250 mg/kg. *Baccharis dracunculifolia* is broadly used in traditional medicine in Brazil, in inflammatory and gastrointestinal diseases. Although the total extract was inactive, the isolated the triterpenoid 2 showed anti-malarial and anti-leishmanial activity. *In vitro* screening of substances isolated from the Brazilian folk medicines identified neosergeolide 3, from *Picrolemma spruce,* which inhibits K1 with an impressive EC_50_ = 2 nM; and, the aryltetralone 4 from *Holostylis reniformis*, with an EC_50_ = 20 nM; both are claimed to have good therapeutic window against hepatocytes. Further testing of these compounds would be needed to assess their strengths and weaknesses. The 4-nerolidylcatechol 5 was isolated from another traditional Amazonian treatment of malaria, *Piper peltatum* and shown to have an EC_50_ between 50 and 830 ng/ml. Catechol 5 was also independently isolated from *Pothomorphe peltata* as shown in Table 1.

### Medicinal chemistry

**Figure 5:**
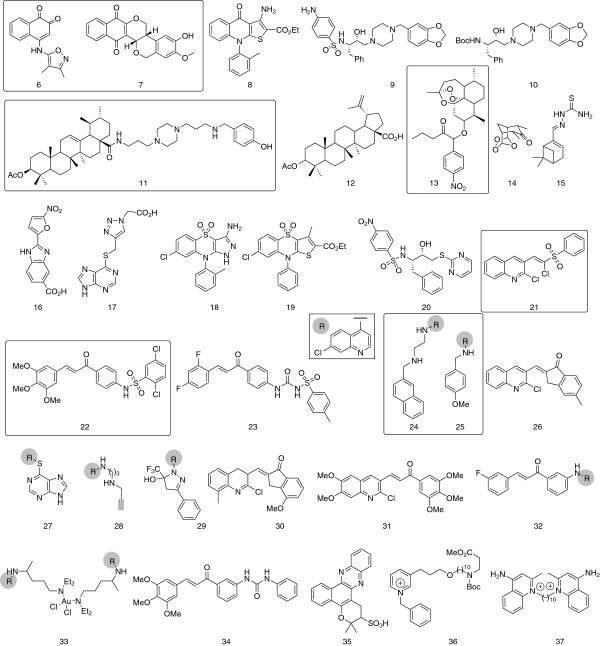
**Structures of compounds with relevant endpoints.** Compounds with the square brackets show activities (EC_50_) below 1 μM.

Medicinal chemistry approaches start from the knowledge of a structure combined with biological activity. Such starting points can be found from screening efforts (for example, pharmaceutical diversity or natural products against a biochemical target or whole cell), *de novo* design or from a published active, which can then act as a starting point for optimization. The molecules that have been identified from various sources against malaria with relevant endpoints and published within the South American medicinal chemistry community are summarized in Table 2, and in addition, their structures are presented in Figure 5. Studies characterizing the spectroscopy of previously described molecules, or studies on marketed anti-malarials have not been included.

**Table 2:** Summary of medicinal chemistry studies

**Compound**	**Authors**	**Target**	**Chemical class**	**Lowest IC**_**50 **_**(strain)**
**6**	**Sperandeo and Brun [**[66]**]**	-	Pyrazolylnaphthoquinones, 5-aminoisoxazole	0.11 μg/mL (K1)
**7**	**Silva *et al. ***[67]	-	1,4-naphthoquinones	0.03 μM (FcB1)
**8**	**Charris *et al. ***[68]	β-haematin	Thieno(2,3-b)quinoline	74.42%^1^
**9**	**Cunico *et al. ***[69]	Aspartyl protease	Hydroxyethylpiperazines	4.6 μM (3D7)
**10**	**Cunico *et al. ***[70]	Aspartyl protease	Hydroxyethyylpiperazines	5.1 μg/mL (W2)
**11**	**Gnoatto *et al. ***[71]	β-haematin	Piperazine, 3-acetylursolic acid	0.08 μM (FcB1)
**12**	**de Sá *et al. ***[72]		Betulinic acid	5.99 μM (W2)
**13**	**Pinheiro *et al. ***[73]	-	Sesquiterpenes	0.05 ng/mL (*P. falciparum* mefloquine resistant)
**14**	**Barbosa *et al. ***[74]	-	Ozonides	13.6 μg/mL
**15**	**Oliveira *et al. ***[75]	Cysteine proteases	Semicarbazone, Thiosemicarbazone	7.2 μM (W2)
**16**	**Camacho *et al. ***[76]	β-haematin	Benzimidazole-5-carbohydrazides	8.43 μM
**17**	**Corrales *et al. ***[77]	Hypoxanthine-guanine phosphoribosyltransferase	6-thiopurine Steroids	82%^1^
**18**	**Barazarte *et al. ***[78]	β-haematin	Pyrazolo and pyrimido benzothiazine dioxide	92.32%^2^
**19**	**Barazarte *et al. ***[79]	β-haematin	Benzothiazines	78.17%^2^
**20**	**Vellasco Junior *et al. ***[80]	Aspartyl protease	Thioetherhydroxyethyl, Sulfonamides	15 μM (W2)
**21**	**Dominguez *et al. ***[81]	β-haematin	Chlorovinyl sulfones	0.025 μM (W2)
**22**	**Dominguez *et al. ***[82]	β-haematin	Sulfonamide chalcones	0.48 μM (W2)
**23**	**León *et al. ***[83]	Glucose metabolism β-haematin	Sulfonylureas	1.2 μM (W2)
**24**	**Arancibia *et al. ***[84]	β-haematin	Rhenium bioorganometalics, Aminoquinoline	0.048 μM (3D7)
**25**	**Rojas Ruiz *et al. ***[85]	β-haematin	Aminoquinolines, thiazolidinone	0.25 μM (3D7)
**26**	**Charris *et al. ***[86]	β-haematin	E-2-quinolinylbenzo-cycloalcanones	90%^3^
**27**	**Vashist *et al. ***[87]	β-haematin	Quinolone, 6-thiopurine	inactive
**28**	**de Souza *et al. ***[88]	β-haematin	4-aminoquinolines, platinum (II) complexes	84 %^4^
**29**	**Cunico *et al. ***[89]	β-haematin	4-aminoquinolines	1.39 μg/mL (W2)
**30**	**Rodrigues *et al. ***[90]	β-haematin	Quinoline	Active
**31**	**Domínguez *et al. ***[91]	Cysteine protease falcipain	Quinolinyl chalcones	19 μM (FcB1)
**32**	**Ferrer *et al. ***[92]	β-haematin	Chloroquinolines	94.93%^3^
**33**	**Navarro *et al. ***[93]	β-haematin	Gold-chloroquine complexes	−^5^
**34**	**Domínguez *et al. ***[94]	Cysteine protease β-haematin	Phenylurenyl chalcones	1.76 μM
**35**	**de Andrade-Neto *et al. ***[95]	-	Naphthoquinones, Phenazines	1.67 μM (W2)
**36**	**Hilário *et al. ***[96]	-	3-alkylpyridines alkaloids	<3.38 μM (W2)
**37**	**Rodrigues *et al. ***[97]	β-haematin	Bisquinoline	56.76 %^2^

^1^ Percentage of inhibition of parasite growth (*P. berghei* in mice) at day 9 (dose: 10 mg/kg).

^2^ Inhibition of globin proteolysis (IGP) expressed as percentage.

^3^ Inhibition of β-haematin synthesis (IβHS) expressed as percentage.

^4^ Inhibition of parasite multiplication on days (dose: 25 mg/kg).

^5^ Activity expressed as IC_50_(CQDP)/IC_50_(complex).

Those compounds having EC_50_ values less than 1 μg/ml (with a square box around) are discussed further as this is the typical potency cut-off required for “Validated Hits” – the starting points for drug discovery projects, as considered by the Medicines for Malaria Venture [98,99].

New pyrazolylnaphthoquinones (heterocyclic naphthoquinones, building on the atovaquone template bearing 3-aminopyrazole rings) and 5-aminoisoxazole analogues showed activity against *P. falciparum*, *Trypanosoma cruzi* and *Trypanosoma brucei*. The 5-aminoisoxazole analogue 6 showed an EC_50_ of 110 ng/mL and an independent naphthoquinone, 7, demonstrated an EC_50_ of 30 nM against FcB1. A novel piperazinyl/steroidal analogue, 11, also inhibited FcB1 with an EC_50_ of 0.08 μM. Pinheiro *et al.* used a multivariate and quantum mechanical method to analyse 15 dihydroartemisinin derivatives and the most potent compound, 13, showed an EC_50_ of 0.05 ng/mL, over 10-fold more potent than the reported values for dihydroartemisinin. Two papers describe chalcone derivatives: the electrophilic chloro-vinyl sulphone 21 showed an EC_50_ of 0.025 μM against W2 and the sulphonamide chalcone 22 showed an EC_50_ of 0.48 μM. Finally, approaches to aminoquinolines identified the derivative 24 with an EC_50_ of 48 nM, against the 3D7 strain; and new heterocyclic hybrids based on the chloroquine and thiazolidinone scaffolds such as 25 have an EC_50_ of 0.25 μM.

## Discussion

In South America, the morbidity and mortality due to malaria is much less significant than in Africa. However, the continent has historically been the source of two of the major classes of drugs against malaria, and the combination of both biodiversity and skilled medicinal chemists could position the continent in a leading position in the search for the new medicines needed for malaria eradication. The current biggest threat in the fight against malaria is the emerging resistance to artemisinin derivatives [100,101]. Artemisinin derivatives within ACT are the most widely used anti-malarials. Even though there has been a concerted attempt to protect them against resistance by banning artesunate monotherapy for uncomplicated disease, the first signs of artemisinin resistance or insensitivity have been described in Cambodia [102] and more recently in Thailand [103]. There is a great need for new combination therapy, replacing the three days’ dosing of ACT with a single dose that also prevents transmission and relapse (in the case of *P. vivax* or *Plasmodium ovale*) [104].

Pharmacognosy has continued to identify new active structures [105]. However, the progress in bringing forward new medicines from these structures and extracts is extremely difficult. Where such molecules are reported to have interesting properties from observational studies, then it is important to confirm these observations in carefully controlled human clinical studies [106]. Also, it is possible that the active principle is a metabolite from the original extract, and so analysis of plasma samples is also important in understanding and identifying the active species [33]. Secondary metabolites are usually thought to play a key role in protection against predators, and therefore could be expected to be cytotoxic. Screening for activity in early safety assays is therefore of paramount concern here. Ultimately the goal of such experiments is to identify new starting points for medicines, similar to the way that quinine and lapachol opened up new fields in previous centuries. Such approaches require long-term commitment, and hence the need to verify the original clinical observations.

The other approach to discover new drugs is to use medicinal chemistry, either with scaffolds already known to be effective against the parasite, or a target-based approach based on structural biology. The results of this survey show that molecules coming from South American programmes are able to demonstrate innovative and active new structures. However over the last five years, the bar has been raised and a new challenge has been set as a result of the success of phenotypic screening. With over 20,000 structures of compounds active against the parasite deposited in the public domain [107,108], it is important to benchmark the successes found in South America against these results. Clearly the prize no longer goes to compounds that simply kill the parasite *in vitro,* but to molecules that have good properties supporting excellent oral administration in patients, or perhaps equal artemisinins in speed of killing parasites [109], or have a very low propensity to resistance generation [110]. In the light of the malaria eradication agenda, it will be important also to know how these new molecules work in the different stages of the parasite lifecycle [111]. A molecule that could be shown to inhibit the dormant liver stages of *P. vivax* would clearly stand out from the crowd [112]. All of the tools are available to enable South American anti-malarial drug research to make these steps forward over the next five years, the challenge will be to put these together, and focus the agenda to the needs of the South American community.

## Conclusion

Malaria continues to be a health issue, particularly *P. vivax* in the Amazon basin, and *P. falciparum* and mixed infections in northern countries. The natural diversity along with the indigenous folk medicines allows a great potential in the treatment and identification of new anti-malarial drugs, as happened with the South American compounds, lapachol and quinine. New molecules are being identified but their optimization for *in vivo* activity has been slow, arguing that more resource needs to be focused in these areas. In addition, the new assays for transmission and relapse of dormant liver stages need to be put into routine practice. If all this is put together, then South America can again play a leading role in the discovery of the next generation of therapeutics against malaria.

## Abbreviations

ACT: Artemisinin-based combinations therapy; API: Annual Parasite Incidence; G6PD: Glucose-6-phosphate dehydrogenase; RAVREDA: Amazon Network for the Surveillance of Antimalarial Drugs Resistance; WHO: World Health Organization; WRAIR: Walter Reed Army Institute of Research.

## Competing interests

The authors declare that they have no competing interests.

## Authors’ contributions

LRC and TS conceived the review. LRC performed the bibliographical research, retrieved the references and drafted the manuscript. TS, MVGL and TNCW critically revised the document. All authors read and approved the final manuscript.
